# Scientific publication activity during COVID-19 shutdown

**DOI:** 10.1007/s00392-020-01707-9

**Published:** 2020-11-25

**Authors:** Michael Böhm, Sebastian Ewen, Armin Schweitzer, Hugo Katus

**Affiliations:** 1 Klinik für Innere Medizin III, Kardiologie, Angiologie und Internistische Intensivmedizin, Universitätsklinikum des Saarlandes, Saarland University, Kirrberger Str. 1, 66421 Homburg/Saar, Germany; 2 Department of Cardiology, Heidelberg University, Im Neuenheimer Feld 410, 69120 Heidelberg, Germany

The COVID-19 pandemic occurred in the metropole area of Wuhan in the Chinese province Hubei with a rapid dissemination of the virus worldwide [1, 2] leading to a pandemic [3]. After an increase of cases in Europe and Germany [4, 5] warnings and recommendations to limit social contacts were issued. According to the fear of infection, there was a reduction of presentation in European and German emergency departments [6], which might have led to a collateral damage of COVID-19 [6].

**Fig. 1:**
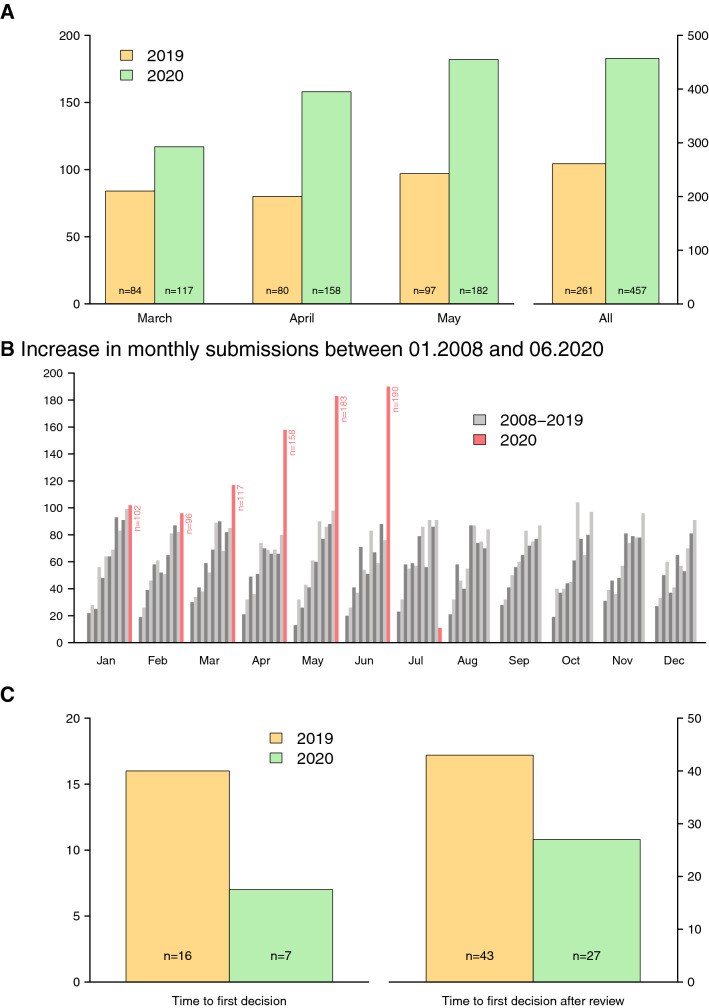
Submission rates of scientific manuscripts (**a**), submission rates over time in comparison to previous years (**b**) and time to final decision of acceptance or rejection (**c**) for Clinical Research in Cardiology

Most congresses like the congress of the European Society of Cardiology, the American College of Cardiology and the German Cardiac Society were cancelled. Furthermore, there were travel restrictions by universities, industry and other healthcare institutions for doctors and scientists. Herein, we report the interesting consequence of travel restrictions and reductions of clinical and scientific meetings including smaller meetings like roundtables and advisory boards. During the three months of shutdown there was an increased submission rate of scientific manuscripts to Clinical Research in Cardiology (Fig. 1a). This trend was stable over time and cannot be explained by an abnormal increase of submission rates to this Journal (Fig. 1b). In parallel, there was a shorter review time and shorter time to decision for acceptance or rejection of journal articles (Fig. 1c). As submissions in 50% of the cases come from other countries, the global shutdown had a positive effect of increased scientific writing activities as well as of accelerated handling of scientific information. This observation cannot lead to the conclusion that a shutdown improves scientific quality or activity but it could be an indication that travel and meeting activities and scientific writing and publishing are competing for time of authors, reviewers, scientists and clinicians.

Herewith, the editors of Clinical Research in Cardiology like to express their gratitude to all authors, reviewers and staff of Springer Scientific Journals for putting so much effort in the generation of rapid review processes, high quality reviews and, thus, important contributions to clinical research and, thus, excellence in patient care.
